# Effect of a relative pricing intervention and active merchandising on snack purchases: interrupted time series analysis of a hospital retailer-led strategy

**DOI:** 10.1186/s12966-023-01426-0

**Published:** 2023-05-04

**Authors:** Catherine L. Mah, Laura Kennedy, Nathan G. A. Taylor, Taylor Nicholson, Emily Jago, Brenda MacDonald

**Affiliations:** 1 Faculty of Health, School of Health Administration, Dalhousie University, Sir Charles Tupper Medical Building, 5850 College Street, 2Nd Floor, 2A03, PO Box 15000, Halifax, NS B3H 4R2 Canada; 2 Nova Scotia Health, Nutrition & Food Services, Room 246, First Floor, Victoria Building, 1276 South Park St, Halifax, NS B3H 2Y9 Canada

**Keywords:** Relative pricing, Consumer food environment, Purchasing, Snacks, Tertiary hospital

## Abstract

**Background:**

Pricing policies have been shown to be an effective lever for promoting healthier dietary choices in consumer food environments. It is not yet well understood how pricing can be used to encourage healthier substitute purchases. The aim of the study was to assess the effect of a retailer-led relative pricing intervention on weekly purchases of targeted snack foods and beverages.

**Methods:**

This was an ecological analysis in a real-world large tertiary hospital consumer food environment setting in urban Canada, comprised of four retail outlets: two large cafeterias, one smaller cafeteria, and one grab-and-go café. An interrupted time series analysis was designed to evaluate the effect of *Snacking Made Simple*, a retailer-led relative pricing intervention applied to 10 popular snack foods and beverages (*n* = 87 weeks, 66 weeks baseline and 21 weeks intervention, April 2018 to December 2019), on weekly purchase differences between healthier and less healthy targeted items, adjusted for weekly sales volume. Five healthier items were price discounted, alongside a price increase for five less healthy items. The intervention was actively merchandised in keeping with behaviour change theory.

**Results:**

Weekly purchases of targeted snacks became healthier during the intervention period (β = 21.41, *p* = 0.0024). This followed a baseline period during which weekly purchases of less healthy targeted snacks had outpaced over time those of healthier targeted snacks (β = -11.02, *p* = 3.68E-14). We estimated that, all else being equal, a hypothetical 9.43 additional weeks of the intervention would be required to transition to net-healthier targeted snack purchases in this environment. The effects of the intervention varied by retail outlet, and the outcome appears driven by specific food items; further, examining merchandising implementation, we posited whether direct versus indirect substitution may have affected purchasing outcomes.

**Conclusions:**

Relative pricing may be a promising way to incentivize healthier substitute purchasing in the consumer food environment. Added attention to merchandising strategy as well as value-add factors within food categories and their effects on price salience may be an important factor in effective intervention design.

**Supplementary Information:**

The online version contains supplementary material available at 10.1186/s12966-023-01426-0.

## Introduction

Poor diet is a leading modifiable risk factor for the global burden of diseases [1], and promoting healthier diets in the consumer environments where food and beverage purchases are made is of growing research interest [2–5]. In particular, pricing interventions have been shown to be an effective lever to promote healthier dietary choices at the point-of-sale [6–8].

Prices are highly influential in consumer food and beverage purchases. Price changes implemented in consumer food environments can affect dietary outcomes both independently and in multicomponent interventions, suggesting they are both necessary and sufficient to alter food and beverage purchases [8, 9]. The behavioural rationale for price changes is to incentivize healthier consumer decision making based on economic constraints and associated motivations for food product selection and demand [10]. Further, psychosocial research has explained how nudge-type merchandising in consumer environments, such as placement, signage, or verbal cues from retail staff, can work adjacent to prices in an integrative fashion to create price ‘salience’ [11–13], increasing the vividness and normative appeal by which consumer goods and their pricing stand out in the marketplace.

Developing effective pricing techniques to promote purchasing is appealing to retailers as part of their business strategy. Retailer-led pricing that promotes healthy diets may be especially attractive and feasible in consumer contexts where maximizing profit is not the leading motivation for food selling, such as public sector organizations (e.g., schools and hospitals) [14, 15]. In these circumstances, the retailer may want to pursue merchandising practices that align with a sense of social responsibility [16]. Retailers in such community contexts may also have stronger awareness of how food affordability and pricing directly affects shoppers in their environments [17].

Although pricing policies are associated with dietary choices at the level of purchases, it is less well understood how they fit into associations with dietary patterns. For instance, sugary drink taxes have been effective in reducing overall demand for sugary drinks in the jurisdictions where they have been implemented [18]. Andreyeva et al.’s recent systematic review and meta-analysis of outcomes following taxation of sugar-sweetened beverages found that these price increases have reduced availability and purchasing of the targeted beverages, however, consistent evidence of substitutions to non-targeted beverages remains elusive [18, 19]. Healthier substitutions are important to a healthier dietary pattern over time [19], and the effect of pricing interventions may be weakened if substitution effects occur encouraging the selection of more attractively priced (e.g., non-taxed) items of poorer nutritional quality [20].

Differential pricing positioning healthier and less healthy items as direct alternatives, is a common occurrence in consumer settings. Recent studies have moreover demonstrated that price promotions tend to favour the selection of foods and beverages of poorer nutritional quality, e.g., through a greater frequency of price promotions for discretionary foods relative to core foods, or a larger proportion of nutrient-poor items in a product category (sweetened beverages) being promoted relative to healthier options (milk, water, 100% juice) [21, 22]. Recent retail environment intervention research has indicated the health promotion value of reversing common merchandising tactics, so healthier items are actively incentivized [23, 24].

Encouraging healthier substitutions in the consumer food environment through relative pricing is complex. Individuals may substitute foods for varied reasons [25], and substitutions can be contingent to price changes (i.e., cross-price elasticity effects) [20]. Substitutions may or may not be associated with complementarity of purchases and can be prompted by point-of-purchase factors in food decision making in real-world settings. Café beverages are a prime example [26]:

Suppose that a compensated price reduction in the price of coffee is experienced by a consumer. Then there exist two kinds of effects which work in opposite directions on the demand for cream. One kind of effect works directly: since the consumer tends to consume coffee and cream together, the demand for cream is increased. The other kind of effect works indirectly via the demand for tea: he now demands less tea, since coffee and tea are substitutes, and less consumption of tea leads to less demand for cream.

In this paper, we will examine how pricing may be used in a real-world consumer food environment setting to encourage healthier substitute purchases. This was an ecological study situated within the pragmatic context of a large tertiary urban health care centre, in the city of Halifax, Nova Scotia, Canada. The main objective was: to examine the effect of a relative pricing and active merchandising strategy on snack food and beverage purchases in the hospital consumer food environment. Specifically, we investigated whether a relative increase in purchases of targeted healthier snacks occurred concurrent to a decrease in purchase of targeted less healthy snacks, in response to the intervention. A secondary objective was to examine how the effect of the intervention on purchasing varied by retail outlet within the hospital. This study contributes to the public sector consumer environment intervention literature, examining active merchandising promoting healthier foods and beverages, where there is a paucity of longitudinal and multi-outlet studies. In addition, our investigation explores determinants of healthier substitute purchase patterns in response to price changes, with a particular focus on within-food category effects.

## Methods

### Study design

An ecological interrupted time series was designed to estimate change in purchases of targeted healthier and less healthy snack foods and beverages over time, in response to a retailer-led relative pricing intervention with active merchandising. Purchasing was examined retrospectively using an administrative point-of-sale (POS) dataset from Nova Scotia Health (NS Health), covering 87 weeks (66 weeks baseline; 21 weeks intervention), from April 2018 to December 2019.

### Public hospital context

NS Health is the health authority for the province of Nova Scotia, the most populous province in Atlantic Canada. NS Health delivers health care services to approximately one million provincial residents and is a leading employer in the region. In 2018, NS Health adopted a Healthy Eating Policy, covering a comprehensive spectrum of services and practice to promote a supportive food environment and healthier diets, and establishing the health authority as the sole retailer in the hospital setting [27]. This means that all retail foodservices outlets in this study are a hospital-run service. Some brand-name franchise items (e.g., coffee) are available, but all suppliers must adhere to hospital policy and procedure including nutrient profiling and pricing. Standard operating procedures (SOPs) were created as guidance for policy implementation. For example, a Retail Food and Beverage Pricing Procedure was developed to support future ad hoc or permanent health-promoting modifications to pricing in the hospital’s retail outlets, as long as minimum operational budgetary criteria were met (i.e., a ‘break-even’ revenue model as compared to ‘maximizing profit’, while modelling a healthier food environment). An authority-wide POS software system was also adopted, making this study possible.

### Intervention approach and setting

In 2019, NS Health designed a retailer-led pricing intervention to encourage a shift in consumer purchasing to healthier snack foods, called *Snacking Made Simple*. Ten popular snack food and beverage items were selected from the regular café and cafeteria menus to be targeted. Five healthier snack items were price discounted, alongside a price increase for five less healthy items (see Table 1). The intended effect was to encourage purchases of healthier snacks, while simultaneously discouraging less healthy alternatives.

**Table 1:** Price, price change, and average weekly purchases of targeted healthier and less healthy snack food and beverage items, during baseline and intervention periods, April 2018 to December 2019

	PRICE			AVERAGE WEEKLY PURCHASES^1^			
	Baseline $CAD	Intervention $CAD	Difference $CAD (%)	Baseline n (SD)	Intervention n (SD)	Difference n	p (adj) **
TARGETED SNACKS^2^							
HEALTHIER							
Apple/Orange^3^	**0.99**	0.75	-0.24 (-24%)	161 (30)	191 (48)	+ 29	0.143
Banana	**0.99**	0.75	-0.24 (-24%)	413 (88)	414 (120)	+ 1	9.84
Bottled Water	**1.95**	1.69	-0.26 (-13%)	683 (94)	662 (94)	-22	3.64
Milk, White SM	**1.25**	0.95	-0.30 (-24%)	175 (30)	166 (26)	-9	2.13
Milk, White LG	**2.00**	1.50	-0.50 (-25%)	166 (29)	180 (27)	+ 14	0.403
LESS HEALTHY							
Loaf Cake Slice	**1.29**	1.59	+ 0.30 (+ 23%)	116 (65)	238 (89)	+ 122	3.59 E-05 **
Mini Cinnamon Bun	**1.99**	2.25	+ 0.26 (+ 13%)	336 (193)	522 (75)	+ 185	8.99 E-08 **
Rice Krispie Square	**1.99**	2.25	+ 0.26 (+ 13%)	256 (85)	149 (83)	-106	0.000134 **
Milk, Chocolate SM	**1.50**	1.89	+ 0.39 (+ 26%)	328 (53)	325 (44)	-3	8.06
Milk, Chocolate LG	**2.25**	2.79	+ 0.54 (+ 24%)	423 (57)	417 (63)	-7	6.66

^1^Average weekly purchases (quantity n, SD) are rounded to nearest integer and hence differences do not sum; differences in average weekly purchases reported as significant ** between baseline and intervention based on multiple tests with Bonferroni correction, α < 0.05

^2^Menu items for which generic names are provided here, correspond to the following items as entered in the hospital point-of-sale (POS) system: Bottled Water = Aquafina B/Water 591 mL; for Milk sizes SM = 237 mL and LG = 473 mL; Loaf Cake Slice = Loaf; Rice Krispie Square = Rice Krispie 50 g

^3^Apple/orange refers to whole apples OR whole oranges sold, as the coding for this ready-to-eat whole fruit item is the same in the point-of-sale system and therefore we are unable to distinguish post-hoc which are apples and which are oranges

The intervention was implemented throughout the Queen Elizabeth II (QEII) Health Sciences Centre, comprised of four retail foodservices outlets in three buildings across two campuses. All outlets are open to hospital staff as well as the public, such as outpatients, families, and visitors. Features of the outlets are described in Table 2 and a map of the retail food outlets within the buildings and corresponding neighbourhood geography is shown in Supplementary Fig. 1.

**Table 2:** Total menu offerings, nutrient profile of menu items with average weekly menu size, and total sales volume, within each retail outlet at QEII Health Sciences Centre, April 2018 to December 2019

	QEII TOTAL all outlets	Large Cafeteria A: Largest cafeteria, full-service kitchen, dine-in	Large Cafeteria B: Large cafeteria, full-service kitchen, dine-in	Grab-and-go Café: Permanent coffee kiosk with no proximal seating	Small Cafeteria: Small cafeteria, limited on-site kitchen prep, limited dine-in
Total menu items over 87 weeks (n)	**568**	500	404	255	216
Menu items over 87 weeks by NS nutrient profile, and average weekly menu size^1^					
MAX n (%)	**134**	122 (24.4%)	101 (25.0%)	68 (26.7%)	66 (30.5%)
Average weekly menu size n	Mean 57	Mean 52.1	Mean 48.7	Mean 24.5	Mean 24.4
		Median 52	Median 49	Median 24	Median 24
		Mode 51, 53	Mode 50	Mode 23	Mode 22
MOD n (%)	**295**	259 (51.8%)	222 (54.9%)	117 (45.9%)	89 (41.2%)
Average weekly menu size n	Mean 95	Mean 70.6	Mean 72.6	Mean 29.3	Mean 26
		Median 69	Median 69	Median 30	Median 26
		Mode 58	Mode 61	Mode 29	Mode 24
MIN n (%)	**139**	119 (23.8%)	81 (20.0%)	70 (27.4%)	61 (28.2%)
Average weekly menu size n	Mean 49	Mean 38.3	Mean 37.9	Mean 27.4	Mean 24.6
		Median 38	Median 38	Median 27	Median 25
		Mode 35, 38	Mode 38	Mode 27	Mode 23
Total sales volume over 87 weeks ($CAD, %)					
$CAD	**$7,319,046**	$3,159,340	$2,443,219	$1,331,161	$385,326
%	**100%**	43.2%	33.4%	18.2%	5.3%

^1^Frequencies and proportions show counts of unique single-serve food and beverage items cumulatively over the 87 week period; average weekly menu size is shown for each grouping of MAX, MOD, and MIN items according to NS Health nutrient criteria, as mean/median/mode menu size per week. Proportions do not sum to 100% due to rounding

Each QEII retail outlet has a comparable main menu with standardized pricing. A key difference between outlets is menu size: a smaller menu is offered at the grab-and-go café and smaller cafeteria, focusing on ready-to-eat items and coffee/tea; at the two larger cafeterias with their full-service on-site kitchens, a larger menu is available with many from-scratch cooked items. Snack foods are a popular purchase, generally consistent across all outlets, and were targeted for the price intervention. Evidence suggests snacks are a useful food category for examining healthier substitute purchasing in the hospital, since they may be discretionary foods, sensitive to price changes in workplaces and schools, and with evidence of substitutive effects in cross-price elasticity studies [20, 28, 29].

### Rationale for price change and active merchandising strategy

In the *Snacking Made Simple* intervention, the five healthier items received a -13% to -25% discount, and the five less healthy items received a concurrent price increase of + 13% to + 26% (see Table 1). The price intervention was consistent at all outlets, which was possible by modifying the prices in the common POS system, at a set time point. The magnitude of price change was based on allowable thresholds for revenue/procurement, and health evidence on pricing typically effective to induce a change in demand. In the literature, +20% is frequently cited as an effective magnitude of price increase to reduce demand for sugary beverages, for instance, but the price changes evaluated across prospective studies has ranged widely; one meta-analysis reported from -10 to -50% for different target items across price discount trials [7]. 

Behaviour change theory around the ‘4Ps’ was used to develop an active merchandising campaign [2]. The goal was to position healthier and less healthy snack items as substitutes, as well as communicating transparently about the health and business rationales for price changes. This was seen as encouraging informed ‘buy-in’ from hospital staff, an internal stakeholder/consumer population with motivation to seek healthier alternatives. Although messaging and visual identity was consistent for the whole of QEII, specific elements of merchandising were implemented dependent on the physical layout features by outlet, which are not uniform. The campaign included: a) Placement (e.g., specialized shelving displays for two of the outlets (Large Cafeteria A and Grab-and-Go Café)); b) Promotion (e.g., key messages, logo, and visual identity used on signage, banners, and staff t-shirts); and c) Training. Specifically, the Placement changes increased the prominence of promoted healthier snacks at the cash register lines, by turning the items towards the traffic flow of customers and increasing the proportion of healthier relative to less healthy snacks. Less healthy snacks were not obscured or hidden, however. The branding (*Snacking Made Simple*) aimed to communicate affordability, convenience, and substitute appeal (see Supplementary Fig. 2 for brand design). Promotional messaging presented the intervention snacks as direct substitutes (healthier versus less healthy), including the idea that at a population-level, the price increase for the less healthy snacks were ‘subsidizing’ the price decrease for the healthier options.

### Measures and analysis

#### Data source and coding

A retrospective longitudinal study was designed to assess change in purchases of targeted snack food items over time in response to the intervention. Baseline sales data was available for 66 weeks and the effect of the intervention was assessed after 21 weeks duration of intervention, a total of 87 weeks of data. This corresponded to a nearly two-year period from April 2018 to December 2019. The emergence of the COVID-19 pandemic in early 2020 resulted in hospital service demands and disruptions that limited time and human resources across all services, including Nutrition. As a result, it was decided that this outcome analysis should be truncated to available interim data, compiled at the end of the calendar year 2019. Administrative sales data was extracted for each of the four hospital retail outlets, provided by NS Health and imported for cleaning and analysis using RStudio Version 1.2.5033 (Orange Blossom).

One research member of the team conducted all cleaning and coding with verification by other team members. Observations were weekly line-item purchases (quantity and $CAD) of single serving units of food and beverage menu items, e.g., apple (unit = single whole fresh apple, loose); small white milk (unit = 237 mL single-serve carton of 2% milk). Purchases were not grouped in ‘baskets’/transactions/per receipt. All food and beverage items were verified as food and beverages, or coded as non-food or taxes and excluded. Items were coded and verified for NS nutrient profile, Canadian Nutrient File food codes, and associated Bureau of Nutritional Sciences (BNS) Food Groups for future studies; nutrient composition data was not used in the analyses in this paper.

### Main outcome and rationale

The main outcome measure was: weekly differences in purchase quantity (units sold) of healthier relative to less healthy targeted snacks, as a discrete variable, adjusted for weekly sales volume (weekly total units of all food and beverage items sold). Hence, weekly values below zero represent less healthy targeted snack purchases exceeding those of healthier snacks; a positive slope over time represents healthier targeted snack purchases outpacing the less healthy targeted snacks in week-to-week time trends.

This outcome measure was seen to be most representative of the head-to-head pricing and merchandising contrast between healthier and less healthy snacks that comprised the intervention. Further, although the 10 targeted snack items remained consistent over the full 87 weeks, dynamic changes in the weekly menu, as well as discontinued and newly introduced menu items, meant the dataset had a great deal of item-level missing data from week to week. As such, using only the targeted snacks in the outcome and adjusting for weekly sales volume as a covariate was well justified. The outcome is a purchase quantity measure (i.e., sales volume as units of food and beverage items sold). It was decided that for the purposes of our study objectives, quantity would be a clearer representation of the data than dollar value ($CAD), given the nutritionally heterogeneous menu with variation in unit costs (e.g., $0.75 CAD for an apple; $7.99 for one portion of stir fry entrée with beef and vegetables, see Supplementary Table 1), which would potentially require post-hoc transformation of the data to establish clinically meaningful values if using dollar value.

### Statistical analysis

Summary statistics during the baseline and intervention periods were calculated for weekly purchases of targeted healthier and targeted less healthy snacks. Scatterplots of weekly purchases and purchase differences were examined over time. The main analysis then used an interrupted time series design, appropriate and feasible given the adoption of the intervention across all sites at a specific time point [30]. Linear segmented regression models were fitted to the weekly purchase ratio data. Data visualization followed Turner et al. [31].

For the main outcome, we fitted the model on the aggregate purchasing across all four QEII outlets. Estimates of both level change and trend change during the baseline (weeks 1–66) and intervention (weeks 67–87) were calculated. The trend effect (beta) during the intervention reflects the adjusted change in purchases over time during the intervention period, relative to what would have been expected to occur if the baseline trend had continued from week 67 onwards, as well as the level change that measures the difference in slopes during the baseline and intervention periods. Final models included total weekly sales volume, measured as quantity of single units of food and beverage items sold per week, as a covariate. For the secondary outcome, we disaggregated by outlet, using Large Cafeteria A as the reference outlet.

A detailed examination of the counterfactual including the effect of seasonality was not possible due to the truncation of the analysis at less than 104 weeks (52 × 2). As an exploratory sensitivity analysis, we compared purchasing in two temporal subsets, matched to the same calendar weeks in subsequent calendar years (weeks 15–35 (2018) and weeks 67 to 87 (2019)).

Sales data for the intervention period was available for 21 weeks; using the effect direction and effect size (slope) during the intervention period, we determined that a transition to net-healthier purchasing would occur at a hypothetical time point past 87 weeks (the end of the data). Based on this determination, using the intercept at 87 weeks and the intervention effect size for the pooled analysis, we estimated the additional duration of intervention required to convert targeted snack purchasing to a net-healthier trend, adjusted for sales volume, all else held equal (weekly purchases of healthier targeted snacks outpacing less healthy targeted snacks, i.e., the ratio for the difference in purchases crossing zero). This calculation assisted with translating significance of time trends for hospital retailing practice, since many consumers in this setting are hospital workers (repeat customers), whose ‘complete’ intervention response may be time-dependent.

Statistical significance was set at α < 0.05. This analysis was part of a larger continuous quality improvement initiative and received an exemption from institutional research ethics board approval.

## Results

### Average weekly purchases during baseline versus intervention

As shown in Table 1, average weekly purchases of targeted snacks differed during the baseline as compared to the intervention period. For three of the less healthy targeted snacks—all three of the baked goods—statistically significant differences in average weekly purchases were detected between baseline and intervention. For the loaf cake slice and mini cinnamon bun, average weekly purchases increased during the intervention period, with loaf purchases nearly doubling. In contrast, there was a significantly lower average weekly purchase of the rice krispie square during the intervention relative to baseline. Of note, the apple/orange was the only healthier item to show a notable magnitude of difference in average weekly purchases (+ 29), however this was nonsignificant after adjusting p-value for multiple tests. Demand for beverage items appeared to be consistent between baseline and intervention periods. The ten targeted snacks comprised approximately 8–10% of all food and beverages purchased weekly during both the baseline and intervention periods; all targeted snacks were offered consistently across all 87 weeks of the data (not shown in data visualization).

In terms of sales volume over the 87 week study period, 568 unique food and beverage menu items were sold across the QEII, as shown in Table 2. This represented a total of $7,319,046 CAD dollar value of purchases made, exclusive of taxes, across all four retail outlets. The distribution of menu items by nutrient profile, and differences in sales volume and menu size as measured in items purchased for the four QEII retail outlets, are shown in Table 2. The two larger cafeterias combined account for nearly three quarters of QEII food and beverage revenues, as measured in dollar value ($CAD). Table 2 also demonstrates, however, that the small grab-and-go café is an important revenue generator and has high sales volume, relative to size of the menu.

### Main outcome: healthier purchases over time during intervention

**Fig. 1:**
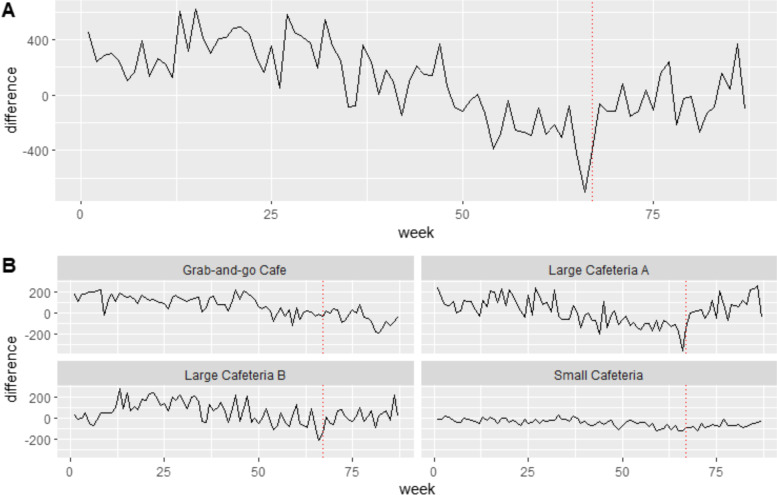
Interrupted time-series showing the impact of a relative pricing intervention on differences between weekly purchases (quantity, n) of healthier and less healthy targeted snack foods at four retail food sites in Halifax, Nova Scotia, from April 2018 – Dec 2019. Baseline = weeks 1–66; Intervention = weeks 67–87, commencing at the dotted line. Panel A shows the aggregate outcomes across all outlets at the QEII; Panel B shows the disaggregated purchases per each of the four retail outlets. All final models were adjusted for weekly sales volume

Outcomes of the main time series analysis can be found in Fig. 1. In the main outcome model (Fig. 1, Panel A), we found that weekly purchases of targeted snacks became healthier during the intervention period (β = 21.41, *p* = 0.0024) after adjusting for total sales volume (unadjusted: β=21.67, *p*=0.00211). This is indicated in the increasing difference in weekly purchases of healthier relative to less healthy snacks, during the intervention from week 67–87. This finding followed a baseline period during which weekly purchases of less healthy targeted snacks had been outpacing those of healthier targeted snacks (β = -11.02, *p* = 3.68E-14) (unadjusted: β=-10.97, *p*=3.97E-14). Differences remained significant when only isolating the temporal subset of baseline corresponding to intervention period by calendar year.

There was a significant level change between baseline and intervention periods (β = -1,343.93, *p* = 0.0118), representing a net shift towards less healthy. It is worth noting that substantial week-to-week fluctuations in purchases (noise) can be seen over time. However, based on the effect direction and size for the positive (healthy) time trend during the intervention period, and the intercept at week 87, we estimated that all else being equal, a hypothetical 9.43 additional weeks of the intervention past week 87 would have been required to transition to net-healthier targeted snack purchases.

### Secondary outcome: variation in time trend by retail outlet

The effects of the intervention varied by retail outlet (Fig. 1, Panel B). Large Cafeteria B as well as the Grab-and-Go Café both significantly exceeded the effect of Large Cafeteria A in their positive trends in purchases of healthier relative to less healthy targeted snacks over time during the intervention.

In contrast, Small Cafeteria did not differ significantly from Large Cafeteria A. Figure 1, Panel B shows through visualization how purchasing at the Small Cafeteria was relatively flat for the targeted snacks throughout the entire 87 weeks of data, during both baseline and intervention periods. Supplementary Figure 3 additionally presents disaggregated time trends in total sales by retail outlet as a dollar value outcome ($CAD), with the intervention period seasonally matched to a temporal subset of the baseline period (calendar year prior); this visualization is intended to support contextual interpretations of the secondary analysis based on quantity.

## Discussion

This paper examined the effect of a relative pricing and active merchandising strategy to incentivize healthier snack food and beverage purchases in a large urban tertiary hospital consumer food environment. Five healthier and five less healthy snack items were selected for a price change, with an accompanying branded merchandising campaign.

The intervention appears to have ‘worked’: over time during the intervention, purchases became relatively healthier, with healthier targeted snack purchases outpacing less healthy purchases in week-to-week trends during the intervention period, adjusted for sales volume. Moreover this was found after a baseline period in which the less healthy snack purchases outpaced the healthier snacks. Based on the effect during the intervention, and the intercept at week 87, we estimated that all else being equal, a hypothetical 9.43 additional weeks of the intervention past week 87 would have been required to transition to net-healthier targeted snack purchases.

The effects of the intervention differed, however, by retail outlet. The largest cafeteria, Large Cafeteria A, appears to have been a driver of the overall trends in purchases, even after adjusting the models for sales volume. Large Cafeteria B and the Grab-and-Go Café appeared to have an even healthier performance than Large Cafeteria A. However, further study of purchasing at the Small Cafeteria would be warranted as a potential outlier in this consumer environment. Although summary measures of purchasing and overall volume (Tables 1 and 2 respectively) should be interpreted with caution given time-varying confounders, it is worth noting that the revenues from Small Cafeteria A are the lowest despite having a menu size that is relatively comparable to the Grab-and-Go Café.

### Differential food and beverage price sensitivities

One important explanation for the findings may be the differential price sensitivity for healthier versus less healthy foods, as well as foods versus beverages, that has been described frequently in the literature. For instance, past retail evidence suggests price elasticity of demand is high for soda but quite low for healthier frequently subsidized foods such as fresh fruits and vegetables [6, 7]. Although purchases became healthier over time during the intervention in this study, this was an ecological finding that may have reflected a wide range of price sensitivity of demand for the targeted snacks, which was a mixed category in this consumer environment that encompassed fresh whole fruits, beverages, and baked goods.

Additionally, since both beverages and foods were targeted within each of the healthier and less healthy groupings, it is possible that heterogeneous price sensitivity for foods versus beverages may have blunted some of magnitude of effect in the time series as well. Although we were not able to quantify the contribution of specific menu items to the outcomes, summary statistics for purchasing during baseline as compared to intervention showed that average beverage demand seems to have been relatively flat throughout (albeit with fairly large standard deviations); only the largest size of chocolate milk showed a small nonsignificant decrease in average weekly purchases.

### From-scratch baked goods: exploring substitution and complementary effects

It should be noted that on average, snacks offered at the QEII tend to have a better nutrient profile than snacks at similar neighbourhood retail outlets nearby in the community. The NS Food and Beverage Nutrient Criteria is a nutrient profiling system for schools and other public organizations in the province that follows a three-tier rating scheme similar to a traffic-light system, where foods and beverages are coded as either MAX-Maximum (most nutrient dense), MOD-Moderate (some essential nutrients), or MIN-Minimum (foods to limit) [32]. Supplementary Table 1 provides a sample menu during the study with corresponding nutrient profiling and unit prices before taxes. The Healthy Eating Policy sets a target healthy menu ratio for the hospital retail outlets by nutrient criteria, set at 80:20, i.e., 80% of offerings are MAX/MOD and 20% are MIN. Some MIN foods are not offered at all, for instance, no full-sugar soft drinks are sold. Other MIN sweetened beverages are available by request (e.g., diet soda, chocolate milk), but not actively merchandised. Wherever possible, snacks are prepared from-scratch rather than by procurement, so that their portion size and nutrient composition can be standardized. For instance, baked goods for the intervention (loaf cake slice, mini cinnamon bun, rice krispie square, see Table 1), were baked on-site from-scratch by recipe to fulfil MOD nutrient criteria per serving unit, and then packaged for sale as ready-to-eat.

Although we cannot directly infer the contribution of specific food items to the analysis, the baked goods stood out in terms of summary differences between baseline and intervention periods. Some research on psychosocial determinants of consumption suggests that from-scratch cooked foods hold value to consumers in ways that is associated with perceived nutrient profile. This suggests that from-scratch could be an important residual confounder in assessing purchasing in this study and any future substitution analyses. From-scratch foods may be perceived as ‘less processed’ and hence ‘healthier’ relative to a pre-packaged counterpart [33]. At the QEII, demand appears to have gone up significantly during the intervention period for the loaf cake slice and mini cinnamon bun; and down for the rice krispie square. On the whole, we saw a ‘healthy’ trend during the intervention period in the time-series, an ecological effect. Yet it does seem that demand for certain, ‘less-healthy’ baked goods may have increased.

In retrospect, we posited whether this within-category finding may have been driven by price salience effects: higher prices are often expected for from-scratch baked goods, such as the mini cinnamon bun, as a foodservices value-add process. The ‘mini’ portion size could also be viewed by consumers as conveying cuteness and hence desirability, or even a healthier portion size. This may have increased demand for one of the targeted ‘less healthy’ snacks despite the higher prices during the intervention, even potentially reinforced through price salience based on the healthy snacking merchandising messages.

In contrast, demand for the rice krispie square appears to have gone down during the intervention—and from-scratch may be an explanation here as well. Hoenink et al. [20], in an examination of uncompensated cross-price elasticities within-category from their Price ExaM intervention study [34], found that cheaper (but equally nutrient-poor) home-brand foods were readily substituted for name-brand foods in specific food categories: beverages and snacks. The divergent purchasing for rice krispie squares, in contrast to the other baked goods, suggests that rice krispie squares baked from scratch may have been viewed as relatively similar to a pre-packaged equivalent, and hence not worthy of the price increase—yet exactly the same price increase levied on the cinnamon bun. ‘From-scratch cooking’ and other value-add processes and claims in foodservices consumer categories are worth further study in the promotion of healthier substitutions through price. The baked goods in our study were all positioned and promoted as ‘less healthy’ targeted snacks in this intervention despite considerable attention to their preparation on-site from-scratch, including portion controls. This may have influenced purchasing outcomes in response to price, as well as merchandising (price salience) effects [11].

### Dose of the intervention

The magnitude of price change is a key aspect of the ‘dose’ of intervention received in consumer food environments [34, 35]. However not as well detailed in the pricing literature are other variables that comprise ‘dose’ within a complex retail setting. The optimal proportion of price-targeted foods within a given menu or menu size, for instance, has not been established. Furthermore, it is not well understood how price changes may be perceived differently within multi-level interactions in the store (e.g., magnitude of price change as interacting with: consumer price sensitivity [36]; customer time spent such as a lengthy wait at the cash register; or overall traffic at the store level). Our time series analysis adjusted for weekly sales volume, but could not account for many of these other factors.

The noisier patterns of purchasing at the Large Cafeterias, as compared to the small Grab-and-Go Café, as well as the relatively flat purchasing at the Small Cafeteria over time, suggest that further study is needed to explore important covariates interacting with the prices encountered by consumers. For instance, Grab-and-Go Café is situated in a central, high-traffic area close to hospital parking and a variety of clinical inpatient and outpatient services. It is well known for being the ‘coffee spot’ and its sales may reflect a focus on beverages accordingly. Grab-and-Go Café is located within the same building as Large Cafeteria A. It would be expected that hospital staff may purchase coffee from Large Cafeteria A at mealtimes, then at the Grab-and-Go Café throughout the rest of the day.

### Strengths

This paper featured key strengths. First, real-world hospital administrative sales data was used through collaboration between researchers and decision makers. This was a pragmatic study design and the findings have direct applicability to health services and continuous quality improvement [27]. The data was generally well suited for an interrupted time series analysis, addressing calls for greater longitudinal studies in retail food environment interventions [15]. In addition, the availability of multiple retail outlets within the QEII location allowed us to explore both the global and disaggregated effects among retail outlets, a gap previously identified in single-outlet price studies. Third, the presence of a supportive institutional context in terms of the overarching Healthy Eating Policy was seen as a strength.

### Limitations

We note the following limitations. First, this was an ecological study of purchasing data. As such, it was not possible to examine substitution directly without receipt-level (basket) data. Although we adjusted for sales volume within both the aggregate as well as outlet-disaggregated models, we did not further evaluate the contribution of specific food items to the time trends, as there were too many targeted items to be able to reasonably accommodate each as a covariate with appropriate interaction terms. In addition, because interrupted time series are limited in addressing time-variant confounding, a valuable next step in the research, should it be feasible in light of hospital quality improvement, would be a quasi-experiment integrating a comparison group (e.g., permitting other modelling such as difference-in-differences to address time-varying confounders). Our examination of differences in purchasing trends among the four retail outlets as a secondary analysis was an essential step towards setting parameters for a comparison group design, by demonstrating that a ‘parallel trends’ assumption among the retail outlets in a single health sciences centre cannot necessarily be presumed. Second, it should be noted that the magnitudes of change in purchasing as a level change as well as a slope change were relatively small. On the one hand this could be attributed to ‘dose’ as described above, where only ten snack items were selected for intervention targeting, comprising approximately 10% or less of weekly menus. Third, this retailer-led intervention encompassed diverse food categories under a single nutrition promotion merchandising campaign, which meant, given known evidence from price interventions, potentially competing cross-price sensitivities affecting findings; for instance, future analyses would benefit from disaggregating beverages from the other items. Fourth, we suspect that an important source of autocorrelation would be the differential consumer use of the four retail outlets, which by virtue of their co-location within the QEII may be frequented for reasons that contribute to temporal as well as spatial autocorrelation.

## Conclusion

Prices are an influential determinant of purchasing and are an important lever for health promotion in consumer food environments. The analysis in this paper demonstrated that a relative price change in combination with active merchandising resulted in healthier targeted snack purchasing trends over time. The results disaggregated by four retail outlet types as well as the inclusion of sales volume as a time-varying covariate suggests differential purchase outcomes for different outlets. This study has also contributed to evidence on how the response to pricing interventions promoting healthier substitute purchases may be partially explained by variation in active merchandising implementation influencing price salience, as well as differential price sensitivity for different foods and beverages within the snack category based on value-add attributes.

## Supplementary Information

**Additional file 1: Supplementary Table 1.** Nutrient Profile of Menu Items from QEII Retail Foodservices Outlets, 2019. List of items on offer during a single week atone retail outlet with corresponding food group and nutrient coding including provincial nutrient profiling system (NS Food and Beverage Nutrient Criteria, 2016) and retail price. **Supplementary Figure 1.** Map of QEII Health Sciences Centre. Map not to scale; shows the buildings within the corresponding physical campuses and neighbourhood geography. Retail outlets in this study are located in buildings 1a (Large Cafeteria A; Grab-and-Go Café), 3 (Small Cafeteria), and 9 (Large Cafeteria B). Reproduced with permission from QEII Foundation 2022 https://www.nshealth.ca/sites/nshealth.ca/files/qeii-building-finder-map-colour.pdf. **Supplementary Figure 2.** Snacking Made Simple Merchandising Campaign Branding at Nova Scotia Health, 2019. **Supplementary Figure 3.** Interrupted time-series showing the impact ofa relative pricing intervention on total sales revenues ($CAD) at four retailfood sites in Halifax, Nova Scotia, from April 2018 – Dec 2019. Baseline = weeks 1-66;Intervention = weeks 67-87, commencing at the dotted line. Shading indicates atemporally matched subset corresponding to the calendar year segment during andprior to the intervention, during baseline.

## Data Availability

The data in this study comprise hospital administrative data and are not publicly available. Derived data supporting the findings of this study are available from the corresponding author upon request and with permission of Nova Scotia Health.
