# Supplementary material for: Effect of a relative pricing intervention and active merchandising on snack purchases: interrupted time series analysis of a hospital retailer-led strategy
Source: Int J Behav Nutr Phys Act. 2023 May 4;20:56. doi: 10.1186/s12966-023-01426-0 (PMC10158715; doi:10.1186/s12966-023-01426-0)
Supplement: Supplementary file 1 — Additional file 1: Supplementary Table 1. Nutrient Profile of Menu Items from QEII Retail Foodservices Outlets, 2019. List of items on offer during a single week atone retail outlet with corresponding food group and nutrient coding including provincial nutrient profiling system (NS Food and Beverage Nutrient Criteria, 2016) and retail price. Supplementary Figure 1. Map of QEII Health Sciences Centre. Map not to scale; shows the buildings within the corresponding physical campuses and neighbourhood geography. Retail outlets in this study are located in buildings 1a (Large Cafeteria A; Grab-and-Go Café), 3 (Small Cafeteria), and 9 (Large Cafeteria B). Reproduced with permission from QEII Foundation 2022 https://www.nshealth.ca/sites/nshealth.ca/files/qeii-building-finder-map-colour.pdf. Supplementary Figure 2. Snacking Made Simple Merchandising Campaign Branding at Nova Scotia Health, 2019. Supplementary Figure 3. Interrupted time-series showing the impact ofa relative pricing intervention on total sales revenues ($CAD) at four retailfood sites in Halifax, Nova Scotia, from April 2018 – Dec 2019. Baseline = weeks 1-66;Intervention = weeks 67-87, commencing at the dotted line. Shading indicates atemporally matched subset corresponding to the calendar year segment during andprior to the intervention, during baseline. [file 12966_2023_1426_MOESM1_ESM.docx]

SUPPLEMENTARY TABLES

Supplementary Table 1

**Nutrient Profile of Menu Items from QEII Retail Foodservices Outlets, 2019.** List of items on offer during a single week at one retail outlet with corresponding food group and nutrient coding including provincial nutrient profiling system (NS Food and Beverage Nutrient Criteria, 2016) and retail price.

| Food Group^[[1]](#footnote-1)^ | PRODUCT  CATEGORY^[[2]](#footnote-2)^ | Menu Item^[[3]](#footnote-3)^ | CNF Code^[[4]](#footnote-4)^ | NS NUTRIENT CRITERIA (MAX/MOD/MIN)^[[5]](#footnote-5)^ | Price (retail) |
| --- | --- | --- | --- | --- | --- |
| baked dessert | Recipe | Whl. Grn. Date Sqr. | 4081 | MIN | 2.25 |
|  | Ready-to-eat | Pie | 3941 | MIN | 1.99 |
| bottled water | Ready-to-eat | Montellier Carbonated. Water | 2918 | MOD | 1.99 |
| bread | Ready-to-eat | WW Bread 2Slc | 7193 | MAX | 1.29 |
|  | Ready-to-eat | Izzy`s Bagel with Butter or Margarine | 3671 | MIN | 1.95 |
| cereal | Ready-to-eat | Cold Cereal with 250ml Milk |  | MOD | 3.29 |
|  | Ready-to-eat | Cereal Box Kelloggs |  | MOD | 0.99 |
|  | Ready-to-eat | Zing Cereal Bowl |  | MOD | 4.99 |
| egg dish | Recipe | Egg, boiled | 130 | MAX | 0.99 |
|  | Recipe | Egg, fried | 129 | MAX | 1.99 |
| fruit | Ready-to-eat | Banana | 1704 | MAX | 0.75 |
|  | Ready-to-eat | Apple | 7216 | MAX | 0.75 |
|  | Ready-to-eat | Fresh Cut Pineapple | 1734 | MAX | 2.29 |
| fruit drink | Ready-to-eat | Dole Juice  450 ml | 7419 | MIN | 1.95 |
|  | Ready-to-eat | Tropicana 236ml | 6470 | MAX | 1.69 |
| granola bar | Ready-to-eat | Nutrigrain (fruit filled) | 6595 | MIN | 1.09 |
|  | Recipe | Rice Krispie 50g | 4887 | MIN | 2.25 |
|  | Recipe | Overnight Oats | 5143 | MAX | 4.99 |
|  | Ready-to-eat | Instant Oatmeal, maple and brown sugar, dry | 1421 | MIN | 1.29 |
| milk flavoured | Ready-to-eat | Milk Chocolate  237 ml | 69 | MIN | 1.89 |
| milk unflavoured | Ready-to-eat | Milk White  237 ml |  | MAX | 0.95 |
| mixed-dish (non-plant protein) | Recipe | Lasagna | 6742 | MOD | 4.99 |
|  | Recipe | Stir Fry, beef, vegetables | 6769 | MOD | 7.99 |
|  | Recipe | Shepherd Pie | 6773 | MOD | 5.99 |
|  | Recipe | Asian Dumplings | 6607 | MIN | 4.99 |
|  | Recipe | Meatballs w/Rice | 7424 | MIN | 5.99 |
| mixed dish (burger) | Recipe | Chicken Burger, lettuce tomato and mayonnaise | 6339 | MOD | 4.29 |
|  | Recipe | Hamburger, large, single patty, condiments, vegetables | 6239 | MOD | 3.10 |
|  | Recipe | BBQ Double Slider, double patty, plain | 4637 | MOD | 7.00 |
| mixed dish (chicken) | Recipe | Chicken Quesadilla | 7102 | MOD | 5.26 |
|  | Recipe | Stirfry, chicken, vegetables | 6770 | MOD | 7.99 |
|  | Recipe | Pad Thai | 6762 | MOD | 7.99 |
|  | Recipe | Chicken Pot Pie/Sweet Potato Biscuit | 4965 | MIN | 6.99 |
|  | Recipe | Chicken Enchilada Combo, rice | 4951 | MOD | 9.49 |
| mixed dish (fish) | Recipe | Baked Haddock | 3199 | MOD | 5.49 |
|  | Recipe | 1 Fish Cake w/ Chutney | 6719 | MAX | 2.75 |
| mixed dish (non-meat) | Recipe | Rice | 4523 | MOD | 1.99 |
|  | Recipe | Stuffing | 3740 | MOD | 1.99 |
|  | Recipe | Vegetable Samosa (per person) | 6751 | MIN | 9.99 |
|  | Recipe | Moroccan Stew & Rice | 5545 | MOD | 6.49 |
|  | Recipe | Stuffed Bell Pepper |  | MOD | 4.29 |
| mixed dish (plant protein) | Recipe | Chickpea & Curry |  | MOD | 7.29 |
|  | Recipe | Chickpea Curry Bowl |  | MOD | 7.29 |
|  | Recipe | Baked Beans | 3248 | MOD | 3.32 |
|  | Recipe | Side Beans |  | MOD | 1.99 |
|  | Recipe | Curry Vegetable Chickpea |  | MOD | 7.29 |
| Muffin/biscuit | Recipe | Mini Cinnamon Bun | 7088 | MIN | 2.25 |
|  | Recipe | Muffin, carrot | 6631 | MOD | 1.35 |
|  | Recipe | Banana Bread | 3688 | MIN | 1.50 |
| pasta | Recipe | Daily Pasta w/Multigrain Roll |  | MOD | 5.99 |
|  | Recipe | Daily Pasta/Whole Fruit |  | MOD | 5.99 |
| pizza | Recipe | Pizza, vaggie, cheese | 6428 | MIN | 3.79 |
|  | Recipe | Pizza, pepperoni, cheese | 6429 | MIN | 3.99 |
| pudding | Ready-to-eat | Pudding | 4237 | MIN | 1.99 |
|  | Recipe | Rice Pudding | 4247 | MIN | 3.99 |
| salad | Recipe | Chicken Caesar Salad | 6739 | MOD | 7.49 |
|  | Recipe | Taco Salad | 6410 | MOD | 3.99 |
|  | Recipe | Garden Salad | 6737 | MAX | 3.99 |
|  | Recipe | Side Greek Salad | 6738 | MAX | 2.49 |
| sandwich/wrap | Recipe | Breakfast Sandwich, egg, cheese, bacon | 4551 | MIN | 3.49 |
|  | Recipe | Egg Salad Sandwich, homemade | 6747 | MOD | 4.29 |
|  | Recipe | Tuna Salad Sandwich | 6749 | MOD | 4.49 |
|  | Recipe | Egg Salad Multigrain | 6747 | MOD | 3.49 |
|  | Recipe | Chicken Club Sandwich, grilled chicken, bacon, tomato, lettuce and mayonnaise | 6753 | MIN | 6.99 |
|  | Recipe | Ham & Cheese | 4642 | MOD | 4.99 |
| soda | Ready-to-eat | Pop 591 ml | 5288 | MIN | 2.10 |
| soup | Recipe | Turkey Vegetable | 958 | MOD | 7.99 |
| sushi | Recipe | Sushi, vegetable, rice | 6764 | MIN | 6.49 |
|  | Recipe | Sushi, fish, rice | 6763 | MIN | 6.99 |
| sweetened beverage | Ready-to-eat | Iced Tea | 4908 | MIN | 3.19 |
| vegetable drink | Ready-to-eat | V8 juice | 5586 | MIN | 3.00 |
| yogurt beverage | Recipe | 16 oz Smoothie, various fruit flavours | 6993 | MOD | 3.99 |
| yogurt | Ready-to-eat | Blbry Oikos Yog. | 7471 | MOD | 1.99 |
|  | Ready-to-eat | Mixed Berry & Yogurt Parfait | 6821 | MOD | 3.49 |

SUPPLEMENTARY FIGURES

Supplementary Figure 1

**Map of QEII Health Sciences Centre.** Map not to scale; shows the buildings within the corresponding physical campuses and neighbourhood geography. Retail outlets in this study are located in buildings 1a (Large Cafeteria A; Grab-and-Go Café), 3 (Small Cafeteria), and 9 (Large Cafeteria B).

Reproduced with permission from QEII Foundation 2022 <https://www.nshealth.ca/sites/nshealth.ca/files/qeii-building-finder-map-colour.pdf>.


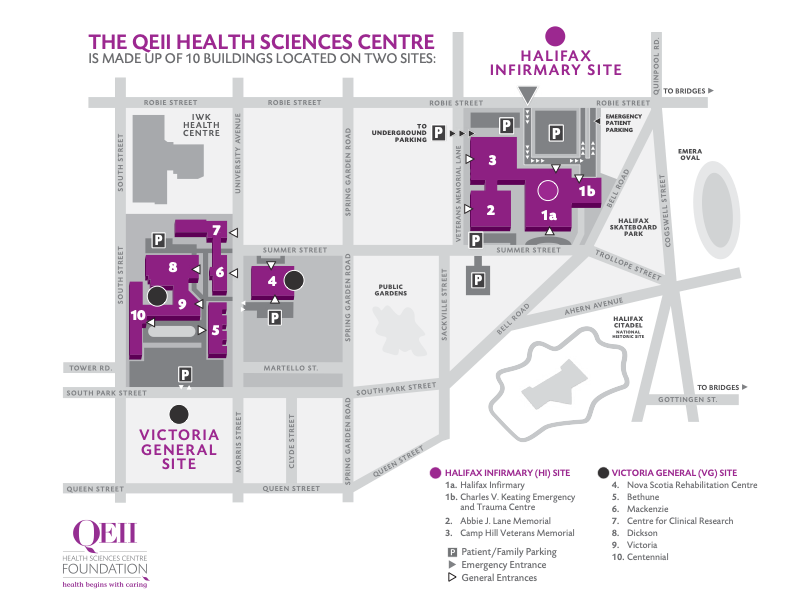


Supplementary Figure 2

***Snacking Made Simple* Merchandising Campaign Branding** at Nova Scotia Health, 2019.


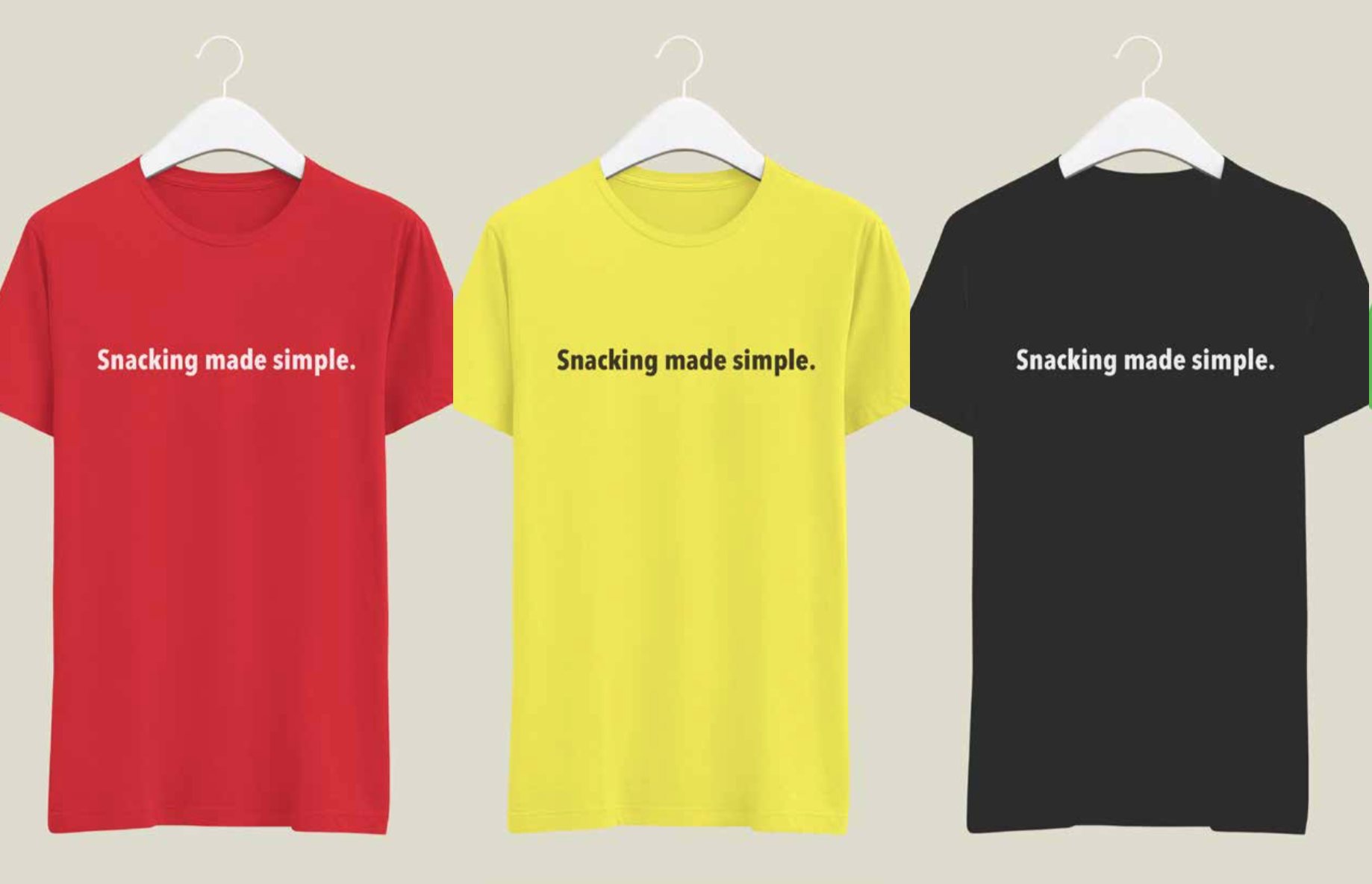

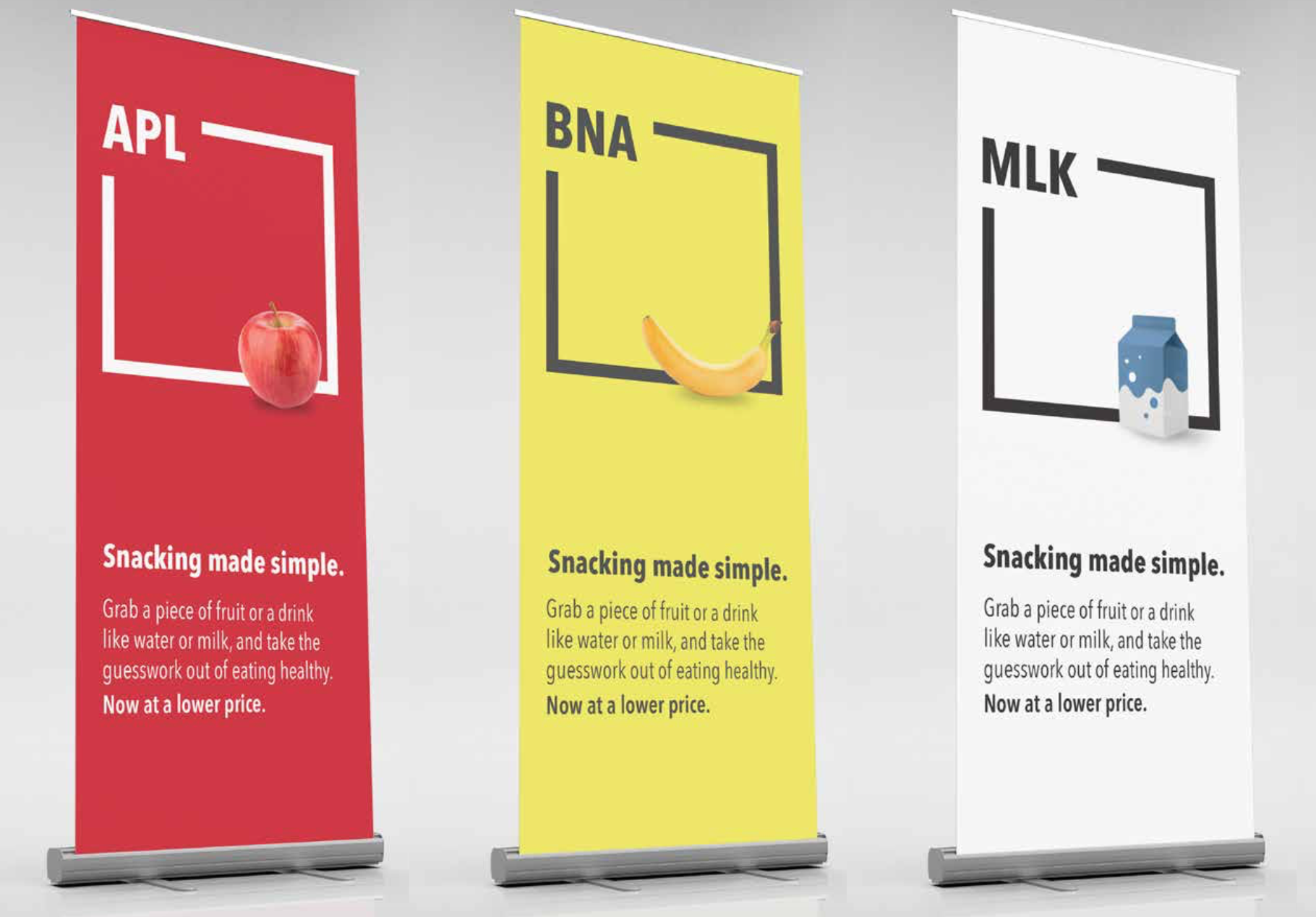


Supplementary Figure 3

**Interrupted time-series showing the impact of a relative pricing intervention on total sales revenues ($CAD) at four retail food sites in Halifax, Nova Scotia, from April 2018 – Dec 2019.** Baseline = weeks 1-66; Intervention = weeks 67-87, commencing at the dotted line. Shading indicates a temporally matched subset corresponding to the calendar year segment during and prior to the intervention, during baseline.


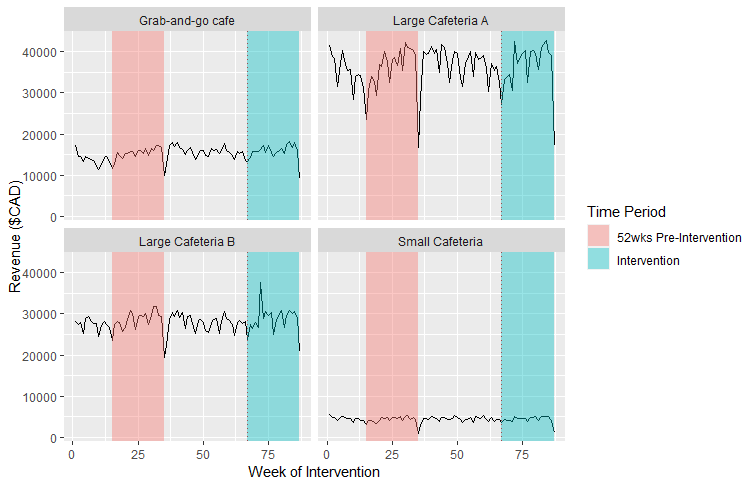


1. Food group classifications based on: Kirkpatrick SI, Raffoul A, Lee KM, Jones AC. Top dietary sources of energy, sodium, sugars, and saturated fats among Canadians: Insights from the 2015 Canadian Community Health Survey. Applied Physiology, Nutrition, and Metabolism. 2019;44(6):650-8. [↑](#footnote-ref-1)
2. Recipe indicates an on-site hospital kitchen-prepared item; ready-to-eat refers to an item procured by the hospital as ready-to-eat for retail sale. Ready-to-eat also includes items such as bottled beverages or whole fruit that can be sold as ready-to-eat without further transformation at the hospital kitchen. Most recipe offerings are portion-controlled for foodservices management (i.e., reducing wastage) as well as nutrient profiling purposes (i.e., to meet nutrient/gram weight serving criteria thresholds). Use of recipes is consistent with commercial kitchen practice and hence items may be pre-packaged on-site in the single serve portion. [↑](#footnote-ref-2)
3. Item names are reproduced as entered in the POS system software. [↑](#footnote-ref-3)
4. Canadian Nutrient File (CNF) food code (<https://food-nutrition.canada.ca/cnf-fce/index-eng.jsp>); some mixed dishes and specialty items are not presented with a corresponding food code here. [↑](#footnote-ref-4)
5. Nutrient profiling as coded to NS Food and Beverage Nutrient Criteria. [↑](#footnote-ref-5)
